# School-Based Tobacco Control and Smoking in Adolescents: Evidence from Multilevel Analyses

**DOI:** 10.3390/ijerph17103422

**Published:** 2020-05-14

**Authors:** Seong Yeon Kim, Myungwha Jang, Seunghyun Yoo, Jung JeKarl, Joo Youn Chung, Sung-il Cho

**Affiliations:** 1 Department of Public Health Science, Graduate School of Public Health, Seoul National University, Seoul 08826, Korea; sykimh37@gmail.com (S.Y.K.); syoo@snu.ac.kr (S.Y.); 2 Institute of Health and Environment, Seoul National University, Seoul 08826, Korea; mwjang9@gmail.com; 3 Department of Health Convergence, College of Science and Industry Convergence, Ewha Womans University, Seoul 03760, Korea; ablajung@naver.com; 4 Korea Health Promotion Institute, Seoul 04554, Korea; claire.jy75@gmail.com

**Keywords:** adolescent, smoking, school-based tobacco control, social norm

## Abstract

Since 2015, universal comprehensive school-based tobacco control programs have been provided in all primary and secondary schools in Korea. This study explored the association of school-level tobacco control with adolescent smoking, and the interactions to investigate whether gender moderates the impact of school tobacco control programs and school-level norms. Both school- and individual-level data were drawn from the 2015 School-Based Tobacco Prevention Program Survey. Multilevel logistic regression analyses were performed using data from 4631 students (ages 10–18 years) who were nested in 62 secondary schools in Seoul, Korea. Students who participated in more prevention programs were less likely to smoke (OR = 0.47, 95% CI 0.30–0.74). The effect of the programs was significantly moderated by gender. For boys, exposure to a greater number of programs decreased the risk of smoking (OR = 0.32, 95% CI 0.18–0.57) but not for girls. At the school level, the school norm regarding tobacco control regulations was negatively associated with smoking (OR = 0.28, 95% CI 0.11–0.76), and its effect was significant for girls only (OR = 0.35, 95% CI 0.17–0.76). This study highlights how the school environment is associated with adolescent smoking behavior, and the effects of programs and norms are different by gender. The findings suggest the need to develop strategies to enhance school-based tobacco control programs and the school norm considering gender differences.

## 1. Introduction

Globally, tobacco use is the leading cause of preventable death and remains a major public health challenge. The tobacco industry targets adolescents and uses aggressive marketing tactics to induce young people to experience tobacco products, increasing the risk of detrimental health consequences [1,2]. Hence, to curb the tobacco epidemic, many countries have enhanced tobacco control intervention to reduce smoking initiation during adolescence [3].

The school environment has been a focus for intervention in adolescent health behaviors [3,4,5,6]. Adolescents spend a large part of their time at schools, where they form and reinforce their attitudes, beliefs, and social norms regarding tobacco use and other health-related behaviors [7,8,9,10]. Several theories of health behavior, including triadic influence theory, support that adolescent smoking behavior is not only influenced by the proximal social context but also by distal contexts, such as schools [11,12]. As social influences of the school environment have received considerable attention, school-based smoking prevention interventions have been developed based on an ecological approach [13,14]. To date, numerous school-based tobacco control interventions have been developed, and many studies have examined their effects on adolescent tobacco use. However, the effectiveness of school-based interventions is largely unclear. Some previous studies suggested that interactive social influences programs have long-term effects on smoking prevention [15,16]. Similarly, a recent Cochrane review showed positive effects of school-based interventions at the longest follow-up [3,17]. However, another review suggested the effect was inconclusive [18]. In addition, regarding school tobacco policy (STP), a study showed that the effects of a policy alone is weak [19]. Similarly, the policy with environmental and educational interventions did not show significant effects [20]. However, a recent review pointed that STPs can be effective, and their impact depends on comprehensive implementation of policy [21]. Due to these mixed findings and the lack of long-term evaluations of school-based tobacco control, the overall effects of such interventions are unclear [1,18].

In Korea, with enactment of the National Health Promotion Act in the 1990s and ratification of the Framework Convention on Tobacco Control (FCTC) in 2005, the government has reinforced tobacco control policies, including both price and non-price policy [22]. To prevent youth smoking, school-based tobacco control programs were implemented in 1999. However, by 2014, only about 10% of the schools in Korea were smoke-free schools and officially supported by the government [23]. With support of the Ministry of Health and Welfare, the smoke-free schools were obliged to conduct smoking prevention programs and promote tobacco control environments; otherwise, it was left to the schools’ autonomy. However, with a significant tobacco tax increase in 2015, the total budget for school-based tobacco control programs, which was 5.6 billion won (about 5 million USD) in 2014, increased to 44.4 billion won (about 40 million USD) in 2015, and universal comprehensive school-based tobacco control programs have been provided in all primary and secondary schools. Schools implement tobacco control activities, such as lectures, counseling, and anti-smoking campaigns, or obtain support from the Office of Education, and public health centers. Through various efforts in Korea, the prevalence of adolescent smoking (smoked at least once in the past 30 days) has declined considerably for decades, from 13.3% in 2007 to 6.7% in 2018, and the rate of students experiencing school-based tobacco control programs per year has increased steadily from 57.5% in 2014 to 72.5% in 2018 [24]. Despite the remarkable change achieved over a decade, the decrease in the smoking rate has been almost stagnant for boys, while the smoking rate in girls increased slightly over the last 4 years.

Social norms have become a major focus of public health interventions. Several theories of behavior, such as social norms theory and social learning theory, support the influence of social norms on the individual health behavior [25]. Social norms have been explicated at both the individual and social level [26,27]. In general, social norms at the individual level represent perceived norms, which refer to individuals’ perceptions about others’ behaviors and attitudes, whereas at the social level, it represents a social entity’s code of conduct [26,27]. School-level norms, such as norms of disapproval toward tobacco use, reflect the overall attitude of students toward tobacco use in a school [7,26]. School-level anti-smoking norms can provide opportunities or protective barriers for students, and thereby can influence the smoking behavior [7]. Previous studies demonstrated that smoke-free school policies have an effect on the students’ perceived anti-smoking norms to some extent [28,29]. Moreover, some prior studies examined the effect of school-level norms regarding adolescent smoking. School-level norms of smoking disapproval showed a significant effect on decreasing adolescent smoking [7]. In contrast, school-level perceived peer tobacco use did not show a significant effect on smoking behavior [30]. However, few studies have considered the social norm regarding tobacco control policies.

Studies have found that gender can interact with other contextual factors and moderate their effects on adolescent tobacco use [30,31]. Gender is a key determinant of tobacco use, but the difference by gender was underexplored regarding tobacco control interventions. Some prior studies identified that males and females can respond differently to tobacco control interventions [32,33]. Regarding the school context, Guindon’s study demonstrated that more students who had learned about the smoking harmfulness at the class level decreases the risk of smoking susceptibility for girls only [31]. Similarly, Grogan showed that other students’ negative attitudes toward smoking were stronger predictors of girls’ smoking compared to boys [34]. Girls are more sensitive to social influences and more influenced by extrinsic motivators, such as others’ opinions compared to boys [34,35]. Based on the different gender responsiveness, prior studies have highlighted problems with gender-blind applications of tobacco control interventions [34,35]. Although the school-level norm is an important indicator of the school environment, which plays a critical role in controlling adolescent smoking behavior, knowledge of the interaction effect of the school-level norms and gender on smoking is limited.

To address these issues, this study conducted multilevel analyses to provide evidence supporting the development of adolescent tobacco control interventions by examining how the school tobacco control programs and school environments affect adolescent tobacco use. This study examined the main effects of school programs and school-level norms related to adolescent smoking. We also examined two interactions to investigate whether gender moderates the impact of school tobacco control programs and school-level norms.

## 2. Materials and Methods

### 2.1. Data Source and Study Population

School and individual student data were drawn from the 2015 School-Based Tobacco Control Program Survey, conducted by the Ministry of Health and Welfare and the Korea Health Promotion Institute. This web-based self-administered survey is an annual nationwide cross-sectional survey of adolescents aged 10–18 (grades 5 to 12) nested in primary and secondary schools in Korea. The survey uses stratified three-stage cluster sampling methods and equal allocation methods. Through stratification by geographic area and school type, 1100 primary and secondary schools were randomly selected, and then one classroom for each grade was randomly selected. For each school, the main manager of the tobacco control program conducted the survey about school policy and environments. For students, measures included smoking status, participation in tobacco control programs, and smoking-related attitudes.

The study population was defined as secondary school students living in Seoul, Korea in 2015. From the national survey, a total of 110 primary and secondary schools were sampled in Seoul (10% of 1100 schools). The overall response rate of the sampled schools was 89.1%, and the survey gathered responses from 6858 students. The data from primary schools were excluded from the analyses due to the low smoking prevalence. The analyses included only schools and students matched by school identification. To reduce statistical errors from multilevel analyses, schools with fewer than 30 sampled students were excluded. Overall, the study population comprised 4631 students nested in 62 secondary schools. This study was considered exempt from the requirement for informed consent by the Institutional Review Board of Seoul National University.

### 2.2. Measures

#### 2.2.1. Smoking

This study used the students’ smoking status as the outcome variable. It was measured by the questions “Have you ever smoked a cigarette, even one or two puffs?” and “In the last 30 days, on how many days did you smoke at least one cigarette?”. Students who had ever smoked and smoked at least 1~2 days within the last 30 days were defined as current smokers.

#### 2.2.2. Individual-Level Variables

Gender was included as a dichotomous variable. The experience of witnessing smoking teachers within the school premises was measured by the question “Have you ever seen teachers or school staff smoke within the school premises during the last 30 days?” and was coded dichotomously.

The number of programs that students participated in were included for the analyses. The survey questionnaire enquired about 11 types of activities that students participated in during the study year. The activities include smoking prevention lectures and counseling, field works, anti-smoking campaigns, and so on (Supplementary file, Table S1). To examine dose–response patterns regarding the 11 types of activities, the total number of school-based tobacco control activities was calculated and classified into three groups: 0–1, 2–3, and ≥4.

Individual smoking-related attitudes were measured through a 10-item survey. To examine clusters of intercorrelated items, exploratory factor analyses were conducted and two clustered items were revealed. One cluster was mainly related to tobacco use by themselves and others, while the other was related to tobacco control policies. Therefore, we defined two types of attitudes, attitudes toward tobacco use and attitudes regarding tobacco control regulations (Supplementary file Table S2). Through the analyses, one item with a low factor loading was excluded so that a total of 9 items were used. The individual attitudes toward tobacco use was defined by six items, which involved the perception of tobacco harmfulness, perception of friends’ tobacco use, and intention to smoke themselves. For example, the items include ‘Tobacco is said to be harmful, but in fact it is not that harmful’, ‘I intend to smoke in the future’, and so on. Furthermore, the individual attitude to tobacco control regulations was defined by three items, which include ‘Smoking in public places where many people gather should be prohibited’, ‘adolescent smoking should be prohibited by law or regulation’, and so on. All responses were measured on five-point Likert scales. Furthermore, students’ grades from 7 to 12 was controlled for all analyses.

#### 2.2.3. School-Level Variables

Based on the sex ratio within the school, the school type was classified into boys only, girls only, and co-educational schools. Data regarding the enforcement of a tobacco-free school declaration, the number of staff managing the program, and the number of years as a tobacco-free school were reported by teachers. The enforcement of a tobacco-free school declaration was coded dichotomously for the analysis. The number of staff managing the program was categorized into ‘1′, ‘2–3′, and ‘≥4′; years as a tobacco-free school was categorized into ‘0 year’, ‘1 year’, and ‘≥ 2 years’; and the school smoking rates were classified into ‘0–5%’, ‘5–10%’, and ‘>10%’. 

Since social norms data were not available, we aggregated the data on personal attitudes. Based on Lapinski’s study, some researchers aggregated individual attitude data at cluster level and defined ‘collective attitudinal norms’ as a proxy for social norms [27,36,37]. The individual-level data nested in schools were aggregated, and the school mean smoking rate, collective attitudinal norm toward tobacco use, and collective attitudinal norm regarding tobacco control regulations were calculated. To reduce the variation by school in the multilevel analyses, the school norms were each classified into low (quartile 1), medium (quartiles 2 to 3), and high (quartile 4) groups. A higher value of school norm towards tobacco use means stronger disapproval of smoking, while that of norm regarding tobacco control regulations means more positive norm towards tobacco control regulations. For the contextual interaction analysis, since none of the girls were current smokers within the school with the highest school norm regarding tobacco control regulations (quartile 4), the school norm was reclassified into two groups to reduce the variation: The lowest (quartile 1) and the rest (quartiles 2 to 4).

### 2.3. Analyses

The study analyses were conducted using SAS 9.4. The datasets for schools and students were merged using the school identification number. To construct and categorize the items related to attitudes on tobacco use, an exploratory factor analysis was conducted. Chi-square tests were conducted for descriptive statistics. Using the PROC GLIMMIX statement in SAS, multilevel logistic regression analyses were conducted to examine the association between the school environment and adolescent smoking. For the multilevel analyses, we applied random intercept models, and all continuous individual-level variables were group-mean centered. Model 1 was a null model and was used to examine the intraclass correlation coefficient (ICC), which indicated how much variation in the adolescent smoking status exists between school-level units. The ICC was calculated using the formula ICC = σ^2^_between_/(σ^2^_between_ + $\frac{\begin{array}{c} \pi^{2} \end{array}}{3}$). Model 2 included only the individual-level variables, while model 3 included only the school-level variables. Model 4 applied both individual- and school-level variables. Furthermore, to examine the moderation effects of gender, the interactions for both the number of programs participated in and the school-level norm were applied in model 5. Since only one out of two school-level norms (norm regarding tobacco control regulations) showed significant main effects on adolescent smoking, the cross-level interaction between the school-level norm regarding tobacco control regulations and gender was only examined.

## 3. Results

Table 1 presents the student characteristics and smoking status. Students’ smoking behavior was significantly different by all individual-level factors at *p*-value < 0.0001. In 2015, 8.0% of boys and 2.6% of girls in secondary school were current smokers, and with a higher students’ grade, the proportion of current smokers was increased. About 65% reported that they participated in more than two programs; the proportion of smokers decreased with a greater number of programs. Of the students, 27.8% witnessed teachers smoking within the school premises and 11.2% of those students were smokers. The mean of the individual attitudes toward tobacco use was 27.5, while that of individual attitudes to tobacco control regulations was 11.9. Smokers had lower scores on both attitudes than non-smokers.

Table 2 presents the school characteristics and school smoking prevalence data. A total of 41.9% of the schools had made a tobacco-free school declaration, 38.7% had more than four staff members managing the tobacco control programs, and 30.6% schools had been managing a tobacco-free school for more than 1 year. Of the schools, about 34% had school smoking rates of more than 5%. Regarding school-level norms, the school smoking prevalence was lower in the schools that had more negative norms toward tobacco use and positive norms toward tobacco control regulations.

Table 3 shows the results of two-level multilevel logistic regression analyses. The ICC measured from the null model indicated that schools accounted for 35.8% of the variability in students’ current smoking. Model 2 included all of the student-level variables, and revealed that when students participated in more than four programs, the odds of smoking were significantly lower. Witnessing teachers smoking within the school premises was associated with higher odds of smoking, while both the individual attitudes toward tobacco use and attitudes toward tobacco control regulations significantly decreased the risk. Model 3 incorporated the school-level variables, and revealed that the years managing a tobacco-free school, school smoking rates, and school-level norms were significantly associated with tobacco use in adolescents. After controlling for the individual-level variables, model 4 showed that for schools with a higher smoking prevalence, the odds of a student being a current smoker were significantly higher, whereas for schools with a more positive school norm regarding tobacco control regulations, the odds of a student being a current smoker were lower (odds ratio [OR] = 0.28, 95% confidence interval [CI] 0.11–0.76). By contrast, a tobacco-free school declaration, the school type, the number of staff working on the tobacco control program, the years managing a tobacco-free school, and the school norm toward tobacco use did not show any significant effects on tobacco use.

**Figure 1:**
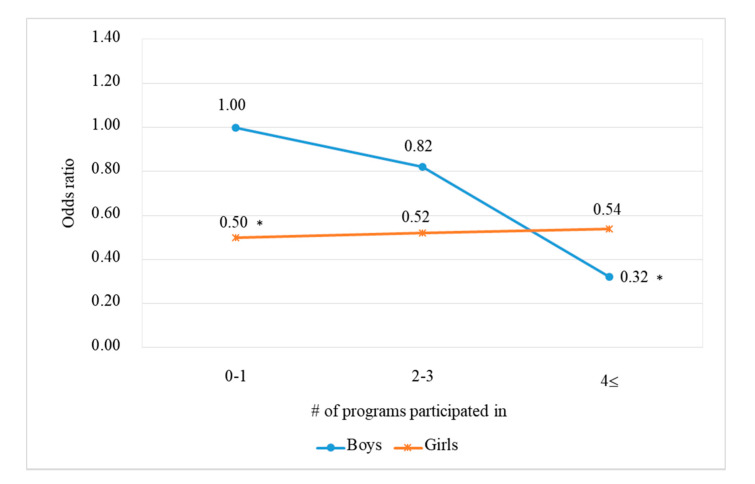
Model-based estimated odds ratio for being a current smoker versus a non-smoker as a function of the number of programs participated in and gender.

Model 5 examined the interactions to identify the moderation effects of gender on the effects of both the school tobacco control programs and the school norm on the adolescent smoking behavior. The interaction term between the number of school programs participated in and gender was significant. In Figure 1, the odds ratios were estimated relative to the reference group of boys who have participated in no more than one school program. For boys, when they participated in more than four programs, the odds of smoking were significantly lower (OR = 0.32, 95% CI 0.18–0.57). For girls, they had a lower odds of smoking than boys (OR = 0.50, 95% CI 0.26–0.95). However, the greater number of programs did not significantly affect the girls’ smoking, and a dose–response pattern was not observed among girls (OR = 0.54, 95% CI 0.27–1.06).

**Figure 2:**
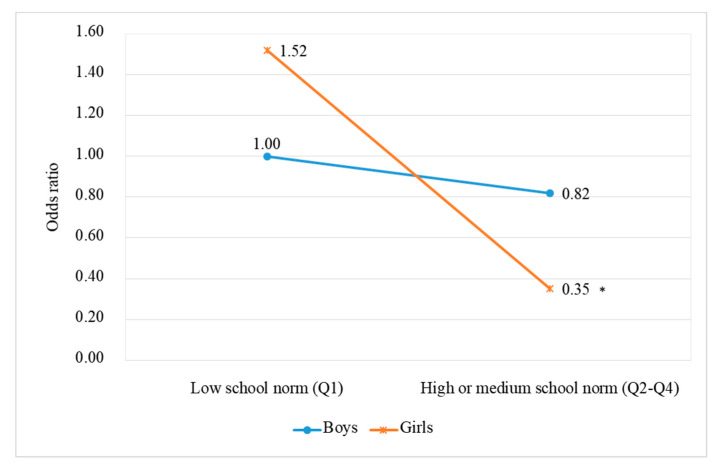
Model-based estimated odds ratio for being a current smoker versus a non-smoker as a function of the school-level norm towards tobacco use and gender.

Furthermore, the effect of the school norm regarding tobacco control regulations on adolescent smoking was significantly moderated by gender (OR = 0.28, 95% CI 0.12–0.65). As Figure 2 shows, a more positive school norm toward tobacco control regulations was associated with a lower odds of smoking among girls (OR = 0.35, 95% CI 0.17–0.76) but was not significantly associated with the odds of smoking among boys (OR = 0.82, 95% CI 0.48–1.40). 

## 4. Discussion

This study evaluated the effects of school-based tobacco control programs and school environments on adolescent tobacco use and the interactions to investigate whether gender moderates the impact of school tobacco control programs and school-level norms. The results indicate that greater exposure to tobacco control programs, and more positive school-level norms regarding tobacco control regulations decrease the risk of smoking, while smoking teachers within the school premises, and higher school smoking rates increase the risk. The effect of the number of programs participated in was significantly moderated by gender, and its effect was greater for boys than girls. Moreover, the effect of the school-level norm on tobacco control regulations was significantly moderated by gender, and its effect was greater for girls than boys.

Regarding the main effect of the number of tobacco control programs on reducing adolescent smoking risk, one study suggested that school programs that involve more than 15 sessions have significant long-term effects on reducing smoking, which demonstrates that ongoing exposure to interventions is needed to prevent adolescent smoking [16]. Similarly, the Surgeon General’s report indicated that programs with more sessions that were sustained for several years were more effective [1]. Since the comprehensive school-based tobacco control programs were implemented in 2015, most of the schools in Korea now provide various types of programs annually, including lectures, consultations, and some experiential programs, such as debates, and tobacco-free campaigns. Although each program might not involve multiple sessions and most are short-term programs, a variety of programs are provided, and students are consistently exposed to tobacco control environments during the school year. A previous study highlighted that comprehensiveness, consistency, and strict enforcement are promising preventive components of tobacco control interventions [19]. Our findings provide additional evidence that consistent and greater exposure to tobacco control programs impacts adolescents’ smoking behavior.

This study also showed that teachers smoking on school premises increased the likelihood of current smoking. Staff who smoke reportedly stimulate the students’ tobacco use, providing cues by smoking on the school premises [38,39]. A recent review suggested that the effects of school tobacco policies on adolescents’ cognitions and behaviors depend on the school context and mode of implementation [21,25]. Inconsistent enforcement of school tobacco policies by staff may lead to a failure of adolescents to internalize anti-smoking personal beliefs [21,40]. Therefore, future school-based tobacco control interventions need to target teachers who smoke.

Furthermore, our findings suggest that not only individual attitudes affect the smoking behavior but also the school-level norm. We examined the effects of two school-level norms on adolescents’ smoking behavior. The present study suggests that the school norm regarding tobacco control regulations has a stronger effect on decreasing adolescents’ smoking. According to Unger, adolescents’ anti-smoking policy awareness and support have strong associations with their smoking behavior and anti-smoking advocacy actions [41]. Furthermore, adolescents’ support for smoke-free public policy decreased smoking susceptibility [13]. In line with previous studies, this study provides evidence that the school-level norms toward anti-tobacco policies affect adolescents’ smoking behavior. When more advocates for tobacco control are present in a school, it can create barriers to adolescent smoking and elicit advocacy actions [7,41]. Individuals are motivated to make positive choices when there is strong social and normative support for healthy choices. Contrary to our expectation, a negative school-level norm towards tobacco use did not show a significant effect on adolescent smoking. However, a prior study showed that school norms of smoking disapproval showed a significant effect on decreasing tobacco use [7]. Since our study used different conceptualizations and measurements of the school norm, the results may be inconsistent. Adolescents are the principal drivers of the tobacco endgame, which aims to halt the tobacco epidemic and achieve a tobacco-free generation. Countries should promote social norms pertaining to smoking more than ever before, because of the influx of new types of tobacco products [42]. Therefore, to increase the effectiveness of tobacco control, schools should target both students and the school environment. Robust regulation and comprehensive tobacco control interventions are needed to strengthen the school-level anti-smoking policy support to help students establish conservative norms and perform advocacy actions against tobacco use [41,43].

By examining the moderation effects of gender, we provide additional evidence that the school tobacco control programs and the school-level norm toward tobacco control regulations have significant effects on tobacco use and show a gender difference in their impact. Regarding the effect of the school programs, the number of programs participated in significantly decreases the risk of smoking for boys, whereas it does not significantly decrease the risk for girls. In contrast, the school-level norm significantly decreases the risk of smoking for girls only. Our findings suggest that boys are more influenced by personal experiences and exposure to the program, while girls are more affected by the surrounding environment. For boys, consistent and greater exposure to tobacco control programs seems more influential compared with girls. Girls are more susceptible to social influences compared with boys [31,44]. For example, girls are influenced more by peers; therefore, peer effects might increase the effects of the school-level norm [33,45]. Previous studies highlighted that tobacco control interventions are gender blind and suggested developing interventions that consider gender differences [34,35]. To curb adolescent smoking, it is important to understand the gender difference and different strategies are needed by gender to develop more effective school-based tobacco control programs. 

One strength of this study was that multilevel modeling allowed us to examine the individual- and contextual-level predictors of adolescent smoking, considering the within- and between-school variation. To our knowledge, this study is the first to examine the interactions of whether gender moderates the effect of school tobacco control programs and school-level norms. However, the study had several limitations. First, the results may not generalize to the national level, because the study sample comprised students and schools from only one city (Seoul). Second, because the study was cross-sectional, causality cannot be inferred. For example, schools with more smoking students may have actively managed the tobacco-free school, thereby causing a reversal effect. Moreover, due to the use of self-report survey methods, smoking prevalence may have been underestimated, especially in girls. The lower smoking rates of girls may have influenced the results. Through this study design, we could not examine the structural association between school tobacco control interventions affecting attitudes and norms, and the attitudes and norms affecting adolescent smoking behaviors. Additional study would need to be done. Regarding types of school, since most of the school sample were co-education schools, the variation by types of school may not be enough to examine the different effects by schools. Additionally, for some programs, whether student participation is voluntary or involuntary is unclear. However, in further analyses, the results were not different, except for activities expected to be voluntary.

## 5. Conclusions

In conclusion, the study examined the first year of the current comprehensive school-based tobacco control intervention. The findings provide evidence supporting the development of adolescent tobacco control interventions by examining how the school tobacco control programs and environments affect adolescent tobacco use. The results indicate that greater exposure to tobacco control programs, and more positive school-level norms regarding tobacco control regulations decrease the risk of smoking. The effects of the programs and the school norm were significantly moderated by gender. This study highlights the need to develop strategies to enhance school-based tobacco control programs and the school norm toward tobacco control regulations considering gender differences.

## Figures and Tables

**Table 1:** Descriptive statistics for individual-level variables.

	Total Sample	Smoking Status		*p*-Value
		Yes	No	
	*n* (%)	*n* (%)	*n* (%)	
All	4631 (100.0)	243 (5.2)	4388 (94.8)	
Gender				
Boys	2260 (48.8)	182 (8.0)	2078 (92.0)	<0.0001
Girls	2371 (51.2)	61 (2.6)	2310 (97.4)	
Grade				
7	945 (20.4)	14 (1.5)	931 (98.5)	<0.0001
8	976 (21.1)	22 (2.2)	954 (97.8)	
9	906 (19.6)	36 (4.0)	870 (96.0)	
10	737 (15.9)	68 (9.2)	669 (90.8)	
11	675 (14.6)	57 (8.4)	618 (91.6)	
12	392 (8.5)	46 (11.7)	346 (88.3)	
# of programs participated in				
0–1	1602 (34.6)	139 (8.7)	1463 (91.3)	<0.0001
2–3	1696 (36.6)	71 (4.2)	1625 (95.8)	
≥4	1333 (28.8)	33 (2.5)	1300 (97.5)	
Witnessed teachers smoking				
Yes	1289 (27.8)	144 (11.2)	1145 (88.8)	<0.0001
No	3342 (72.2)	99 (3.0)	3243 (97.0)	
Attitudes toward tobacco use [Mean (SD)]	27.5 (4.2)	21.6 (5.1)	27.9 (3.9)	<0.0001
Attitudes regarding tobacco control regulations [Mean (SD)]	11.9 (4.0)	9.5 (3.3)	12.0 (4.0)	<0.0001

Percentages are described in rows.

**Table 2:** Descriptive statistics for school-level variables.

	Distribution of School Characteristics	Distribution of Smoking Prevalence in School	
	*n* (%)	Mean	SD
All	62 (100.0)	5.80	9.43
School type			
Boys-only	5 (8.0)	10.66	5.02
Girls-only	13 (21.0)	2.22	3.53
Co-education	44 (71.0)	6.30	10.65
Tobacco-free school declaration			
Yes	26 (41.9)	5.55	7.15
No	36 (58.1)	5.98	10.88
# of staff			
1–3	38 (61.3)	6.35	11.41
≥4	24 (38.7)	4.91	5.01
Years as a tobacco-free school			
0	43 (69.4)	6.75	11.05
1	11 (17.7)	3.86	3.91
≥2	8 (12.9)	3.35	1.52
School smoking rates			
0–5%	41 (66.1)	1.57	1.57
5–10%	11 (17.7)	6.96	1.41
>10%	10 (16.1)	21.83	14.87
School norm towards tobacco use ^1^			
Quartile 1	17 (27.4)	14.94	14.08
Quartile 2	15 (24.2)	3.41	3.27
Quartile 3	14 (22.6)	2.94	2.29
Quartile 4	16 (25.8)	0.83	1.14
School norm regarding tobacco control regulations ^2^			
Quartile 1	17 (27.4)	12.11	15.24
Quartile 2	16 (25.8)	5.25	4.98
Quartile 3	13 (21.0)	3.75	3.37
Quartile 4	16 (25.8)	1.30	3.03

^1^ Collective attitudinal norm towards tobacco use, aggregated from individual reports. ^2^ Collective attitudinal norm about tobacco control regulations, aggregated from individual reports.

**Table 3:** Multilevel analyses: the association of current smoking with individual and school-level factors.

	Current Smoking				
	Model 1	Model 2	Model 3	Model 4	Model 5
Intercept	0.03 (0.02–0.04) *	0.01 (0.01–0.02) *	0.04 (0.02–0.08) *	0.01 (0.01–0.04) *	0.00 (0.00–0.01) *
**Student level**					
Gender					
Boys		1.00		1.00	1.00
Girls		0.42 (0.27–0.66) *		0.71 (0.44–1.14)	0.81 (0.50–1.32)
# of programs participated in					
0–1		1.00		1.00	1.00
2–3		0.85 (0.59–1.22)		0.85 (0.60–1.22)	1.70 (1.06–2.75) *
≥4		0.46 (0.29–0.73) *		0.47 (0.30–0.74) *	1.58 (0.97–2.58)
Witnessed teachers smoking					
No		1.00		1.00	1.00
Yes		2.91 (2.12–4.00) *		2.57 (1.88–3.51) *	2.56 (1.87–3.50) *
Attitudes toward tobacco use		0.57 (0.51–0.63) *		0.56 (0.51–0.63) *	0.56 (0.50–0.62) *
Attitudes regarding tobacco control regulations		0.56 (0.47–0.66) *		0.54 (0.46–0.64) *	0.54 (0.46–0.65) *
**School level**					
School type					
Coeducation			1.00	1.00	1.00
Boys only			0.91 (0.48–1.71)	0.72 (0.32–1.60)	0.64 (0.29–1.39)
Girls only			1.00 (0.49–2.04)	1.20 (0.45–3.22)	1.18 (0.46–3.00)
Tobacco-free school declaration					
No			1.00	1.00	1.00
Yes			1.06 (0.68–1.65)	0.94 (0.54–1.61)	0.99 (0.58–1.68)
# of staff					
1–3			1.00	1.00	1.00
≥4			0.67 (0.41–1.09)	0.69 (0.38–1.24)	0.76 (0.42–1.36)
Years as a tobacco-free school					
0			1.00	1.00	1.00
1			1.34 (0.80–2.27)	1.53 (0.80–2.95)	1.52 (0.80–2.89)
≥2			1.85 (1.01–3.39) *	2.01 (0.99–4.09)	2.15 (1.06–4.34) *
School smoking rates					
0–5%			1.00	1.00	1.00
5–10%			3.20 (1.90–5.41) *	3.84 (2.00–7.39) *	4.16 (2.18–7.95) *
>10%			9.71 (5.03–18.73) *	9.29 (3.57–24.19) *	9.95 (3.91–25.31) *
School norm towards tobacco use ^1^					
Q1			1.00	1.00	1.00
Q2–Q3			0.68 (0.36–1.27)	0.83 (0.38–1.80)	0.87 (0.41–1.86)
Q4			0.36 (0.14–0.90) *	0.50 (0.17–1.46)	0.45 (0.16–1.25)
School norm regarding tobacco control regulations ^2^					
Q1			1.00	1.00	
Q2–Q3			0.71 (0.47–1.07)	0.65 (0.39–1.08)	
Q4			0.32 (0.13–0.75) *	0.28 (0.11–0.76) *	
**Interactions**					
# of programs (2–3) × Gender					1.27 (0.57–2.82)
# of programs (≥4) × Gender					3.33 (1.31–8.48) *
School norm regarding tobacco control regulations^2^ × Gender					0.28 (0.12–0.65) *
School–level random variance	1.8368 *	1.0204 *	0.0739	0.1839	0.1644
AIC	1673.37	1394.18	1593.07	1354.04	1346.81

ICC = 35.8%; * *p* < 0.05; All analyses are controlled for students’ grade. ^1^ Collective attitudinal norm towards tobacco use, aggregated from individual reports. ^2^ Collective attitudinal norm about tobacco control regulations, aggregated form individual reports.

## Supplementary Materials

The following are available online at https://www.mdpi.com/1660-4601/17/10/3422/s1, Table S1: Types of school-based tobacco control activities, Table S2: Exploratory factor analysis of attitudes to tobacco use.
