# Supplementary material for: School-Based Tobacco Control and Smoking in Adolescents: Evidence from Multilevel Analyses
Source: Int J Environ Res Public Health. 2020 May 14;17(10):3422. doi: 10.3390/ijerph17103422 (PMC7277168; doi:10.3390/ijerph17103422)
Supplement: Supplementary file 1 [file ijerph-17-03422-s001.pdf]

Supplementary Materials

Table S1. Types of school-based tobacco control activities.

no.	School-based tobacco control activities
1	Handouts related to smoking prevention
2	Lectures and counseling by teachers
3	Audiovisual education pertaining to smoking prevention and cessation, and the plan of the tobacco industry
4	Discussion regarding the harmful effects of tobacco products and the plan of the tobacco industry
5	Field work such as art contests and quizzes related to smoking prevention
6	Attendance of plays and musicals with a theme of smoking prevention and cessation
7	Peer counseling and mentoring activities
8	Smoking prevention “camp” with family and a teacher
9	Anti-smoking campaigns, including a ban on tobacco sales to minors in the area around the school
10	Establish school tobacco control policies, and implement the tobacco-free school policy
11	Others

Table S2. Exploratory factor analysis of attitudes to tobacco use.

No.	Attitudes to tobacco use	Factor 1	Factor 2	
1	Tobacco is said to be harmful, but in fact it is not that harmful	0.64*	0.06	Attitudes toward tobacco use
2	I think it is fine to smoke one or two times out of curiosity	0.68*	0.07	
3	I feel I want to smoke when I see a smoking scene in movies or magazines	0.81*	0.08	
4	It looks mature when people smoke	0.92*	0.07	
5	It looks cool when a friend smokes	0.90*	0.08	
6	I intend to smoke in the future	0.62*	0.05	
7	Smoking in public places where many people gather should be prohibited	0.07	0.81*	Attitudes regarding tobacco control regulations
8	Adolescent smoking should be prohibited by law or regulation	0.09	0.88*	
9	Education aimed at smoking prevention should begin in elementary school	0.06	0.79*	
